# Bridging the gap: safety and outcomes of intensivist-led ECMO retrievals

**DOI:** 10.3389/fmed.2023.1239006

**Published:** 2023-08-23

**Authors:** Mircea R. Mihu, Marc O. Maybauer, Kaitlyn Cain, Laura V. Swant, Michael D. Harper, Robert S. Schoaps, Joseph M. Brewer, Ammar Sharif, Clayne Benson, Ahmed M. El Banayosy, Aly El Banayosy

**Affiliations:** ^1^Specialty Critical Care and Acute Circulatory Support, Nazih Zuhdi Transplant Institute, Integris Baptist Medical Center, Oklahoma City, OK, United States; ^2^Department of Medicine/Cardiology, Oklahoma State University Health Science Center, Tulsa, OK, United States; ^3^Critical Care Research Group, The Prince Charles Hospital, The University of Queensland, Brisbane, QLD, Australia; ^4^Department of Anesthesiology, Division of Critical Care Medicine, University of Florida College of Medicine, Gainesville, FL, United States; ^5^Department of Anesthesiology and Intensive Care Medicine, Philipps University, Marburg, Germany; ^6^MedStar Heart and Vascular Institute, MedStar Washington Medical Center, Washington, DC, United States

**Keywords:** ECMO retrieval, ECMO transport, ECLS, hub and spoke model, ECPR

## Abstract

**Purpose:**

Most extracorporeal membrane oxygenation (ECMO) cannulations are performed by cardiothoracic surgeons (CTS). Due to an increase in utilization of ECMO and limited availability of CTS, there is a mismatch between ECMO demand and CTS accessibility for remote cannulations. We report our intensivist-led program’s experience in remote ECMO cannulations, retrievals, complications, and outcomes.

**Materials and methods:**

A prospective, single-center, observational study was performed on patients that required ECMO cannulation at the referring facilities and were transported to our institution between program initiation, on October 1, 2014 to September 30, 2022. Results were presented as mean ± SD, median (min – max) or number (%).

**Results:**

Since program commencement, 305 patients were accepted for ECMO retrieval. Three hundred and three patients were placed on ECMO at the 47 referring hospitals among 5 states. In our study, 185 (61%) patients required veno-arterial ECMO and 115 (38%) were placed on veno-venous ECMO. Three patients (1%) were cannulated for veno-arteriovenous ECMO. Twenty patients were cannulated under cardio-pulmonary resuscitation. Most of the patients were transported by ambulance (79%), 14% by helicopter, and 7% by airplane. Six out of the 303 patients did not leave the referring facility. All patients that left the referring hospitals arrived safely to our institution. No major complications occurred in route.

**Conclusion:**

Our study’s findings indicate that non-CTS physicians can successfully cannulate and retrieve patients with a low complication profile.

## Introduction

Patients who develop profound circulatory shock or severe respiratory distress refractory to mechanical ventilation may require extracorporeal life support (ECLS). However, due to its resource-intensive and complex nature, extracorporeal membrane oxygenation (ECMO) is not widely available. Furthermore, previous studies have demonstrated improved outcomes for ECMO patients when they are treated at specialized centers with a high patient volume and expertise (1). Given that a patient’s clinical condition sometimes poses an unacceptable risk for conventional transport, specialized teams from tertiary medical centers travel to referring hospitals to implant ECMO and stabilize the patient for transportation back to the ECMO center for further care. According to the criteria set by the Extracorporeal Life Support Organization (ELSO), this process is referred to as “primary transport” (2).

Considering that over 90% of cardiogenic shock patients experiencing acute myocardial infarction undergo percutaneous coronary intervention outside academic institutions (3), and data from the Society of Thoracic Surgeons in 2016 revealed that more than 70% of coronary artery bypass graft surgeries are performed in low-volume centers, a well-organized regional ECMO center with remote cannulation and retrieval capabilities can provide potentially life-saving interventions to patients treated at smaller hospitals. ECMO retrievals have been conducted for decades, as described in the existing literature (4–9). The feasibility of a retrieval cardiogenic shock team operating within a regional hub-and-spoke model was demonstrated in the cardiac-RESCUE pilot study in France (10). Our center introduced an intensivist-led ECMO retrieval program in 2014, representing the only specialized center capable of remote ECMO cannulation and transport in our state at that time. While most ECMO cannulations are performed by cardiothoracic surgeons (CTS), the increased utilization of ECMO (11, 12) and limited availability of CTS have created a mismatch between the demand for ECMO and accessibility to CTS for remote cannulations, necessitating the involvement of other services to perform cannulations and retrievals. In this report, we present our intensivist-led experience in remote ECMO cannulations, retrievals, and associated complications.

## Materials and methods

A retrospective, single-center, observational study was performed on patients that required ECMO cannulation at the referring facilities and were transported to Integris Baptist Medical Center in Oklahoma City between program initiation, on October 1, 2014 to September 30, 2022.

We included patients treated with veno-arterial (V-A), veno-venous (V-V), and veno-arteriovenous (V-AV) ECMO, cannulated by our physicians or physician at the referring hospitals, and transported to our institution by our ECMO retrieval team. The Integris Baptist Medical Center institutional review board approved this study prior to initiation of this project (IRB # 18–005).

As previously described, our institution offers a 24/7 ECMO retrieval service. Our diverse cardiac intensivist group includes anesthesia, cardiac anesthesia, emergency medicine, pulmonary, cardiology, infectious diseases and medicine trained physicians (13). All members have the ability to cannulate both V-A and V-V ECMO. Our retrieval team is comprised of an ECMO physician, ECMO coordinator, ECMO specialist or perfusionist and a respiratory therapist.

For V-A ECMO cannulations, our practice involves initially attempting to place a 5 or 7 French (Fr) antegrade reperfusion cannula, followed by placing of a 17 Fr, 23 cm long arterial and 23 or 25 Fr venous femoral drainage cannula and initiation of ECLS support. If antegrade cannula placement is unsuccessful, upon return to our institution, we consult cardiovascular or vascular surgery for antegrade catheter placement via cutdown. In some cases, depending on the vessel size, we use 15 Fr or 19 Fr arterial sized cannulas.

For V-V ECMO cannulations performed at outside institutions, we use a dual site cannulation strategy. In the majority of cases, we use 23 or 25 Fr multistage drainage cannula placed via femoral vein and a 17 to 23 Fr short cannula (15 cm length) placed in the right internal jugular vein. In special circumstances (potential bridge to transplant, morbidly obese patients, need for right ventricular support), if the referring hospital has fluoroscopy capability, we use a single-site venous bicaval dual lumen cannula via the right internal jugular vein or a ProtekDuo® (LivaNova PLC, London, United Kingdom) catheter if there is a need for a right ventricular assist device with oxygenator.

The decision to place a patient on ECLS was made by an on-call ECMO intensivist in consultation with the referring physician (cardiothoracic surgeon, cardiologist, pulmonologist, intensivist, or hospitalist). In case of a borderline indication, the on-call ECMO intensivist involved peers on the ECMO team to discuss a specific case. In post-cardiotomy patients we involved our cardio-thoracic surgeon on call in the decision-making process and in patients with acute on chronic heart failure we consulted our heart failure cardiologists to weigh in regarding durable mechanical circulatory support (MCS) or potential transplant candidacy before accepting patients to our institution.

This was a retrospective observational study reviewing patient demographics, body mass index (BMI), indication, location of cannulation, cannulating physician, distance traveled, mode of transportation and complications. Specifically for patients requiring V-A ECMO, we evaluated for the presence of other types of mechanical circulatory support prior to ECLS, acute kidney injury (AKI) prior to ECMO support, the need for renal replacement therapy (RRT). Lactic acid and sequential organ failure assessment (SOFA) score prior to cannulation when available were recorded. Patients with cardiogenic shock stage D and above Society of Cardiovascular Angiography and Interventions (SCAI) classification requiring a minimum of two inotropes or vasopressors were considered for V-A ECMO cannulation (14). For V-V ECMO patients only, days on ventilator, PaO_2_ to FiO_2_ ratio (P/F -ratio) and positive end-expiratory pressure (PEEP) prior to ECLS initiation, as well as the need for inhaled pulmonary vasodilators was recorded. Of note, for V-V ECMO, per our inclusion criteria, eligible patients had a Murray score of 3 or higher or uncompensated hypercapnia with a pH less than 7.2 (Supplementary Table).

Statistical analysis was performed using the SPSS statistical package (IBM, version 26, New York). Results were presented as mean ± SD, median (min – max) or number (%). Differences between groups were analyzed using the independent t-test for continuous variables and Fisher’s exact test for categorical variables. The Mann–Whitney U test was used for variables that did not display a normal distribution. All statistical tests were two-sided, and differences were considered significant when *p* ≤ 0.05.

## Results

**Figure 1:**
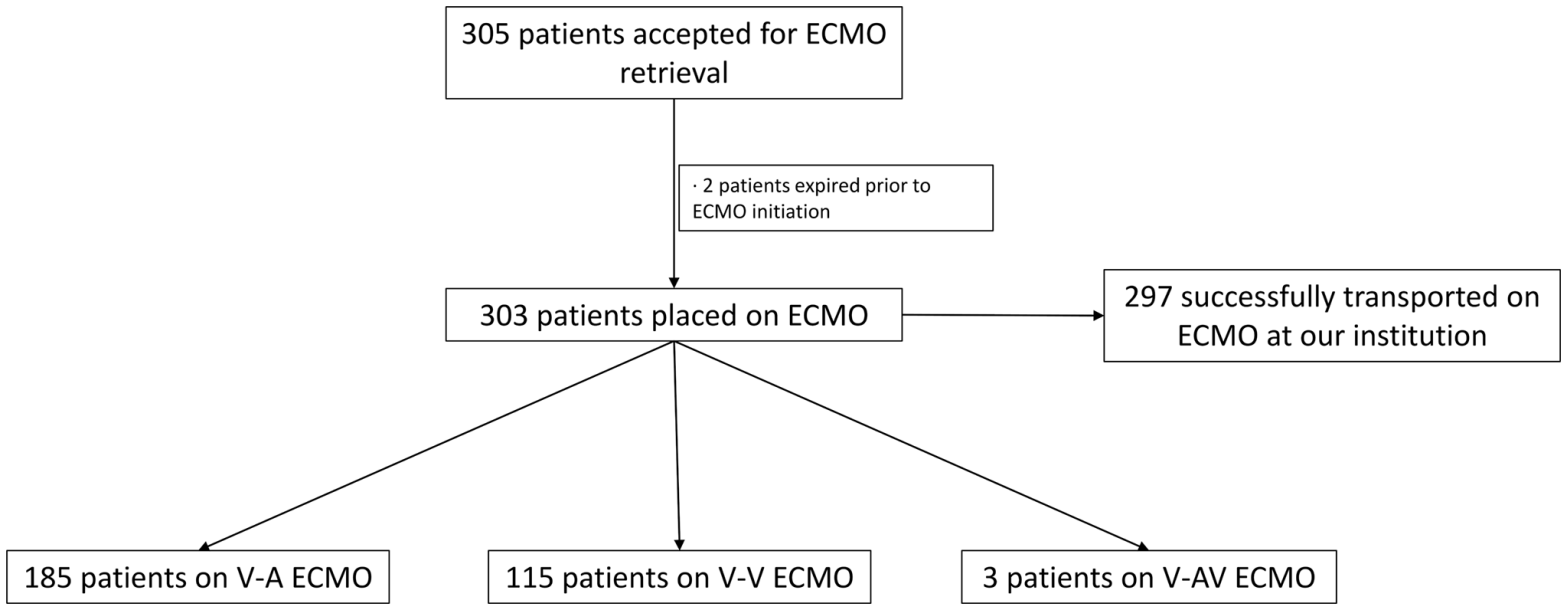
Flowchart diagram of the total number of patients accepted for ECMO retrieval, ECMO type and number of patients transported back to our institution. *ECMO, extracorporeal membrane oxygenation; V-A, veno-arterial; V-V, veno-venous; V-AV, veno-arteriovenous.

Since program commencement, 860 patients were placed on ECMO. Out of the total number of patients placed on ECMO, 305 patients were accepted for ECMO retrieval. Two patients decompensated prior to our team arrival at the referring centers, requiring cardio-pulmonary resuscitation (CPR) and expired prior to ECMO initiation (Figure 1). Since these patients were never started on ECLS, they were excluded from our study.

Three hundred and three patients were placed on ECLS at referring institutions. In our study, 185 patients required V-A ECMO and 115 were placed on V-V ECMO. Three patients (1%) required V-AV ECMO. Our team performed 83% of the total cannulations at the outside hospitals, while 17% were placed on ECMO by physicians at the referring hospitals. All patients that left the outside facility were safely transported to our medical center. The median age was 52 years (18–82), 62% were males and 38% were females. The median BMI was 31.4 Kg/m^2^ (16–84.8) and the median length of stay at the transferring hospital was 3 days (0–104). Sixty six percent (199/303) of cannulations were performed at bedside in the Intensive Care Unit (ICU), while 21% were performed in the operating room (OR) and 13% in the catheterization laboratory. Out of the total number of V-A ECMO cannulations, 20 (11%) were performed under CPR.

The majority of ECMO transports, 79%, were by ambulance, with an average distance of 42.5 miles ±49. Helicopter transport was used in 41 patients (14%), with an average distance of 122 miles ±55. Airplane rescue transport was used in 20 cases (7%) with an average distance traveled of 166 miles ±65.

For V-A ECMO patients, we traveled to 40 centers across 4 states (including our own). The average distance was 58.7 ± 64 miles, with a median of 20 (0.4–299) miles.

We retrieved V-V ECMO patients from 38 centers from 5 states, and we traveled an average distance 66.2 ± 64.6 miles, with a median of 50 (0.4–300) miles.

**Figure 2:**
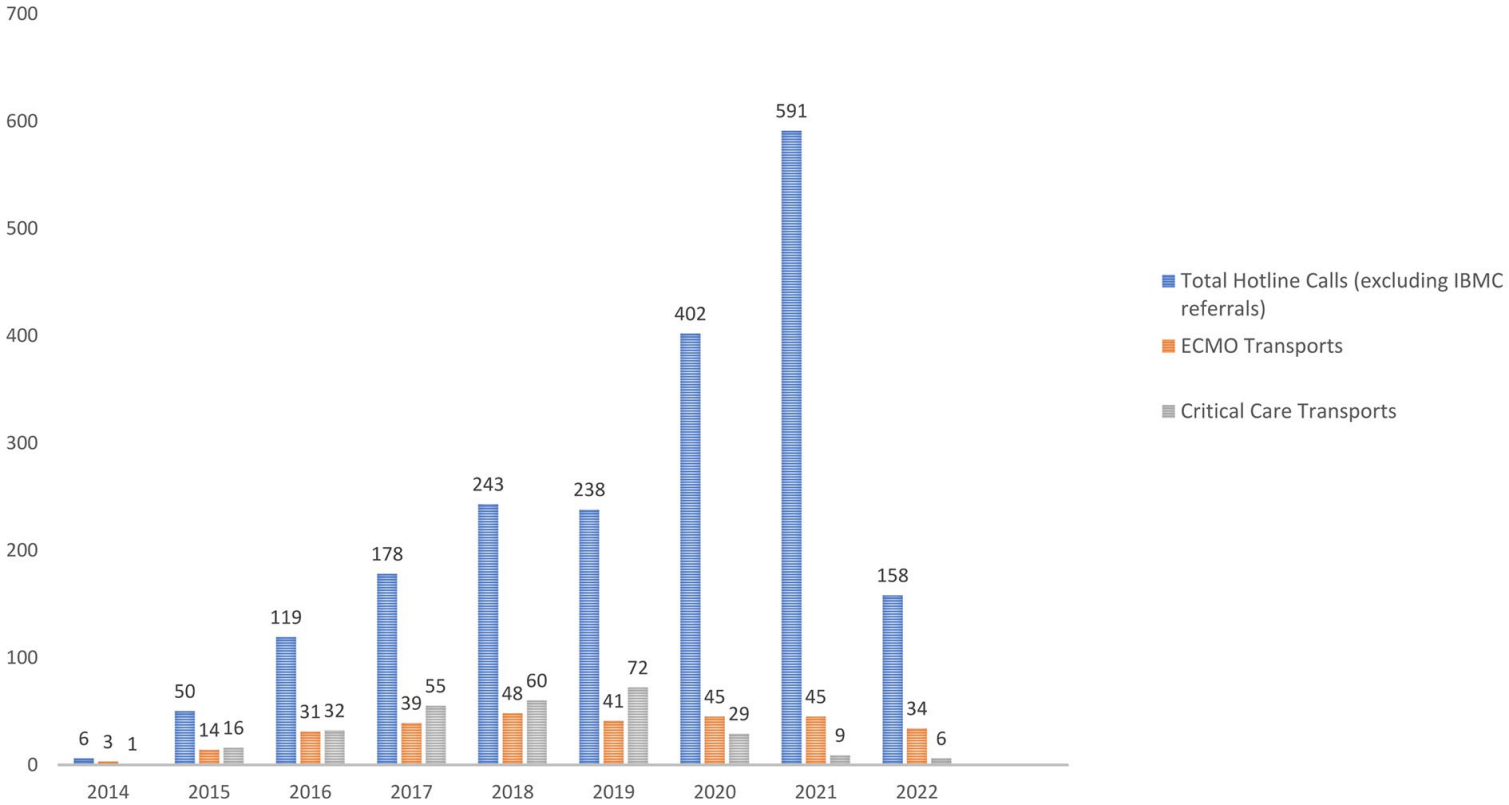
Total number of referrals for ECMO each year, number of patients retrieved on ECMO and number of patients accepted for higher level of care. *Total hotline calls, total number of referrals from outside institutions; *IBMC, Integris Baptist Medical Center; *ECMO transports, number of patients placed on ECMO at the referring institution; *Critical care transports, number of patients accepted for higher level of care and potentially ECMO, deemed stable for transport.

Out of the total number of ECMO referrals we received since program commencement (Supplementary Figure), the acceptance rate (ECMO transports as well as critical care transports) ranged between 51 to 60% between 2014 and 2019 and dropped to 20 and 10%, respectively, during 2020 and 2021. In 2022 we accepted approximately 30% of the total number of referrals (Figure 2).

V-A ECMO was the most common form of ECLS and 185 patients were placed on it. The most common indications for V-A ECMO included post cardiotomy shock in 32.4% of cases followed by cardiogenic shock secondary to myocardial infarction in 19% (Table 1). In patients that required V-A ECMO, 43% (80/185) had other types of mechanical circulatory support prior to ECLS initiation. Intra-aortic balloon pump (IABP) was used in 31% of patients and temporary left ventricular support device (Impella® Abiomed, Danvers, MA) was used in 12% of the cases. The median SOFA score was 13 (7–19) and the median lactic acid was 6.5 mmol/L (0.8–27). Fifty eight percent of patients had developed AKI prior to ECMO initiation and 9% required continuous renal replacement therapy (CRRT). Most of the patients were cannulated peripherally (181/185) and 4 patients were cannulated centrally. About 50% of patients (92) were weaned from ECMO support. The duration of ECLS was 8 ± 8 days and total hospital length of stay was 22 ± 28 days. Limb ischemia was encountered in 24 (13%) patients and cannula site infection in 2 (1%). Nineteen patients required circuit exchange secondary to hemolysis.

**Table 1:** V-A ECMO indications.

V-A ECMO indications	*n* = 185
Post cardiotomy shock	60
Myocardial infarction	35
Post cardiac arrest	27
E-CPR	19
Septic cardiomyopathy	19
Circulatory shock	7
Pulmonary embolism	4
Myocarditis	3
Diabetic ketoacidosis	3
Acute on chronic systolic heart failure	3
Right ventricular failure	2
Post-partum cardiomyopathy	2
Verapamil overdose	1

V-A ECMO, veno-arterial extracorporeal membrane oxygenation; E-CPR, Extracorporeal membrane oxygenation – Cardiopulmonary resuscitation; n, number of patients.

The number of patients that required V-V ECMO was 115. The most common indication for veno-venous ECLS was acute respiratory distress syndrome (ARDS) secondary to coronavirus disease 2019 (COVID 19) pneumonia, which occurred in 45.2% of cases. This was followed by ARDS secondary to bacterial pneumonia in 26% of patients (Table 2). Patients that required V-V ECMO placement had a median P/F ratio of 66 (29–148) and a median PEEP of 14 (5–32). The median days on ventilator prior to ECLS initiation was 4 (0–24) and 9 patients had been ventilated for more than 10 days. The median SOFA score for V-V ECMO patients was 9 and the median lactic acid was 1.9 mmol/L (0.6–10). More than half of the patients (52%) were on inhaled pulmonary vasodilators at the time of ECMO cannulation. In terms of cannulation strategies, 81 were placed on dual site ECMO and 34 had double lumen cannulas. Seventy seven patients were weaned from ECMO. The duration of ECLS was 20 ± 20 days and total hospital length of stay was 40 ± 34 days. Only 4% of the V-V ECMO patients required circuit exchange secondary to hemolysis. No cannula site infection was observed.

**Table 2:** V-V ECMO indications.

V-V ECMO indications	*n* = 115
ARDS secondary to COVID 19 pneumonia	52
Bacterial pneumonia	30
Viral pneumonia	10
Aspiration pneumonia	8
Status asthmaticus	3
Post cardiotomy respiratory failure	2
Cardiogenic shock with associated respiratory failure	2
Respiratory failure secondary to interstitial lung disease	2
Fungal pneumonia	1
Polytrauma	1
Diffuse alveolar hemorrhage	1
Pre Lung-transplant	1
ARDS secondary to pancreatitis	1
Acute respiratory failure secondary to interstitial emphysema and pneumomediastinum	1

V-V ECMO, veno-venous extracorporeal membrane oxygenation; ARDS, acute respiratory distress syndrome; COVID 19, Coronavirus disease 2019; n, number of patients.

Six out of the 303 patients (2%) did not leave the referring hospital. Three patients were successfully placed on veno-arterial ECLS, but after ECMO initiation, they were not able to be stabilized enough for transport therefore care was withdrawn at the referring facility. One patient was placed on V-V ECMO and upon initiation of support patient developed cardiac arrest and resuscitative measures were unsuccessful. One patient that was cannulated by the referring physician, had cannula dislodgment upon transferring to the transport stretcher and expired prior to transportation. One patient cannulated during CPR was initiated on ECLS but shortly after initiation it was realized that patient was on V-V ECMO support. Arterial cannulation was attempted but unsuccessful and the patient expired.

All patients that left the referring hospitals arrived safely at our institution. No major complications or adverse events in route were noted. Specifically, no cannula dislodgment, air embolism, emergent circuit exchange, pneumothorax or cardiac arrest was encountered in route. During transportation, patients required active vasopressor adjustment, volume resuscitation and blood product transfusion. Serial arterial blood gases were performed routinely in route. Notable events included a helicopter emergent landing due to helicopter malfunction in one instance, one ambulance breakdown, an emergent ambulance stop at a rural hospital to replace oxygen tanks and a ventilatory failure. Patient related complications included one ventricular fibrillation episode in a patient supported with V-A ECMO that required defibrillation.

Fifty-five patients required at some point during their hospitalization ECMO reconfiguration or revision. Most of these patients, 30, required hybrid ECMO with different type of configurations. Cannula site change was performed in 12 patients, 4 were converted from V-A to V-V ECMO, 4 patients were converted from V-V to V-A ECMO. One patient had accidental cannula dislodgement during the hospitalization requiring emergent re-cannulation. Three patients were converted to venous-pulmonary artery (V-P) configuration. One patient was converted from peripheral V-A to central V-A ECMO.

V-A ECMO survival to discharge (including E-CPR) was 39.5% and V-V ECMO survival to discharge was 63.5%. All patients (3) that required initial hybrid ECMO (V-AV), expired. Sex, mode of transportation, distance traveled for patient retrieval, cannulator (our group vs. physician at the referring institution) did not show any statistically significant impact on survival. Predictors of survival to discharge for V-A and V-V ECMO are presented in Table 3. For V-A ECMO patients, higher SOFA score and lactic acid were associated with worse outcome (*p* < 0.01 and *p* = 0.03 respectively). Pre ECMO MCS (IABP or Impella) did not contribute to a better outcome (*p* = 0.55). AKI before V-A ECMO initiation was not associated with a worse outcome as well (*p* = 0.55). For patients that required V-V ECMO, younger patients had a higher survival as compared to older ones (*p* < 0.01). P/F ratio, BMI, PEEP, SOFA score, lactic acid, and ventilator days before ECLS support were not associated with significant worse outcome (Table 3). Interestingly, the use of inhaled pulmonary vasodilators was associated with worse overall outcome (*p* < 0.01).

**Table 3:** Predictors of outcome for Veno-Arterial ECMO patients.

Variables	Survivors (*n* = 72)	Non-survivors (*n* = 113)	*p*-value
Age	51.5 ± 15.1	55 ± 15.3	0.13
BMI	32.2 ± 8.9	31.2 ± 7	0.44
SOFA score pre ECMO	12.6 ± 1.9	14 ± 3.1	**<0.01**
Lactic acid pre ECMO	6.6 ± 3.6	8.3 ± 5.6	**0.03**

Predictors of outcome for Veno-Venous ECMO patients.	Variables	Survivors (*n* = 73)	Non-survivors (*n* = 42)	*p*-value
Age	39 ± 13.3	49.8 ± 12.7	**<0.01**
BMI	35.8 ± 10.4	32.8 ± 9.5	0.12
SOFA score pre ECMO	11 ± 3	2.2 ± 1.7	0.30
Lactic acid pre ECMO	2.4 ± 1.9	2.2 ± 1.7	0.54
P/F ratio	73.9 ± 33.2	64.9 ± 19.2	0.08
PEEP	14 ± 5	15 ± 5	0.34
Pre ECMO ventilator days	3.8 ± 5.3	4.3 ± 4.1	0.61

Data expressed as mean ± standard deviation. *p* values ≤ 0.05 were considered significant (in bold).

Age (years); BMI (kg/m^2^), body mass index; SOFA, sequential organ failure assessment; ECMO, extracorporeal membrane oxygenation; Lactic acid (mmol/L); P/F ratio, PaO_2_ to FiO_2_ ratio; PEEP (cmH_2_O), positive-end expiratory pressure.

## Discussion

To the best of our knowledge, this study represents the largest report of an intensivist-led ECMO retrieval program in North America. Our findings demonstrate that non-CTS ECLS cannulations and retrievals are safe, with a comparable complication profile to previously published data (7, 15, 16). It is worth noting that our intensivists undergo comprehensive cannulation training under the supervision of a physician, with a minimum duration of one year, unless they have prior training. Additionally, they are required to perform a minimum of 25 cannulations combined, for both V-A (15 cannulations) and V-V ECMO (10 cannulations).

Out of the total number of patients initiated on ECLS, three patients were unable to be stabilized sufficiently for transport after being placed on ECMO, and care was withdrawn at the referring facility. One patient, cannulated for V-V ECMO due to COVID-19 pneumonia, experienced cardiac arrest and could not be resuscitated, resulting in death. It should be mentioned that during the COVID-19 pandemic, we had strict ECMO inclusion criteria, and therefore V-A ECMO was not attempted in cases of cardiac arrest (17). One patient who underwent CPR was initiated on ECLS, but it was later discovered that the patient was receiving V-V support. Although arterial cannulation was attempted, it was unsuccessful, even with the assistance of a cardiac surgeon, and the patient ultimately passed away. Another patient, cannulated by the referring physician, experienced cannula dislodgement during transfer from the hospital bed to the transport stretcher, which led to their death. This incident occurred during a secondary transport (2) in the early stages of our program, and the security of the cannula was not checked. Consequently, we implemented changes in our practice for both primary and secondary transports, such as adjusting cannula length and increasing the number of sutures required for securement. For dual-site cannulation, we now require a minimum of four sutures per cannula in addition to the securement device, while for dual-lumen cannulas, we require a minimum of five sutures. For femoral arterial cannulas, we preferentially choose the 23-centimeter (cm) length unless the ECMO physician has a specific reason to opt for the 15 cm length.

Having an ECMO retrieval service is crucial for assisting patients at institutions without ECLS capabilities. According to the American Hospital Association, as of 2020, there are over 6,000 hospitals in the US, but based on ELSO data, only 274 centers offer adult ECMO services, and out of those, only 107 have transport capability. Moreover, it is unknown how many centers in the United States have an ECMO retrieval program. Without ECMO support, these patients have an extremely low likelihood of survival, as demonstrated by our data.

ECMO rescue is extremely labor and resource intensive. In our experience, from the time we accept the patient for retrieval, it takes approximately 45 min to 1 h until the team leaves the hospital. Time to destination depends on the distance traveled and mode of transportation. Once at the outside facility, we evaluate the patient at bedside, discuss with the referring provider and obtain consent from the family. This process usually takes around 30 min to 1 h. A V-A ECMO cannulation is performed in approximately 45 min to 1.5 h and V-V ECMO cannulation takes 45 min to 1 h. This time also includes the initial stabilization post cannulation and securing cannulas for transportation. Once the process is completed, we transfer the patient to the transport stretcher and leave the referring facility once the patient is stabilized enough. From the time we accept the patient for ECMO retrieval to the time we leave the referring facility, it takes a minimum of 3 to 3.5 h, excluding the time spent to reach the outside institution.

Out of the 305 patients accepted for ECMO, two patients died before ECLS initiation. Among the 303 patients included in the study, 20 were cannulated during CPR. It is important to note that we presently do not accept ECMO CPR referrals from outside institutions. This means that 22 patients progressed to cardiac arrest from the time of referral to our team’s arrival. However, we were able to stabilize 19 of these patients on ECMO and subsequently transport them to our facility. Furthermore, the median SOFA score for our V-A ECMO patients was 13, and the median lactic acid level was 6.5.

Our complication rates are consistent with other reports published in the literature. Bryner et al. documented four deaths among 195 patients cannulated by their ECMO retrieval team, with four patients dying at the referring hospitals before transport and one patient dying during transportation (7). Foley et al. reported one patient death during cannulation and two deaths before cannulation among 100 patients (15). Fletcher-Sandersjoo et al. observed that two out of 908 patients expired during transportation (16). In another study by Biscotti et al., one patient experienced accidental decannulation of a right internal jugular venous cannula, necessitating the placement of a second cannula in the left internal jugular vein (5). Unfortunately, in our case of accidental arterial cannula dislodgement, the unintended decannulation led to the patient’s demise.

In our data, we are reporting a survival to discharge of 48%. This survival rate is for the combined V-A and V-V ECMO patients with predominantly V-A ECMO patients (61%). The survival-to-discharge rate for V-A ECMO patients was 39% while in our V-V ECMO population was 63.5%. The survival rate for V-A ECMO also includes patients who were not transported back to our institution. Additionally, this survival rate encompasses 20 cases of E-CPR. Of note, our V-A ECMO survival to discharge rate for patients cannulated at our institution is 49% and for our V-V ECMO population during the study period was 59%. The survival rate we report is similar to the data published in the literature. Bryner et al. reported an adult survival-to-discharge rate of 55% in a mixed cohort of 52% V-A ECMO and 48% V-V ECMO population (7). Austin et al. documented a survival-to-discharge rate of 68%, although their analysis included 74% V-V ECMO and only 26% V-A ECMO population. Moreover, the mean age of patients was 40, and the mean SOFA score for V-A ECMO patients only was 10, which is significantly lower than the SOFA score of our population (18). Another study by Brechot et al. focused on patients requiring V-V ECMO retrieval and reported a survival-to-discharge rate of 53.4% (19).

At our institution, we offer both ECMO retrieval and critical care transport services (patients transported by our team under the supervision and care of one of our ECMO physicians). Prior to the COVID-19 pandemic, our acceptance rate ranged from 50 to 60% of total referrals. However, due to bed and staff shortages during the pandemic, the acceptance rate dropped to 10–20%. Although we are gradually witnessing an increase in our acceptance rate this year, the existing challenges mentioned above prevent us from helping as many patients as we were able to before.

As previously described, with the proper infrastructure and a well-trained and experienced team, ECMO retrievals can be safely performed (4–8, 16, 18, 20, 21). It is critical to reach out to and educate providers at smaller, more remote facilities to ensure that patients in dire need, who would otherwise not survive, can receive the necessary assistance and potentially this life saving intervention.

This study is limited by its retrospective nature, being single center, and non-randomized design. Further, randomized, multicenter studies are needed for developing clear strategy for placement and management of patients on ECMO at outside institutions and their safe retrieval.

To our knowledge, this study represents the largest ECMO retrieval study conducted by an intensivist-led ECMO program in North America. In conclusion, our analysis demonstrates that patients who are critically ill with profound shock and/or respiratory failure can undergo safe placement on extracorporeal life support at hospitals without ECMO capability by non-CTS physicians, and subsequently be transferred to larger, more experienced medical centers for ongoing care. It may be beneficial if more centers in North America encourage ECMO retrievals by non-CTS physicians to meet the demand and provide potentially lifesaving intervention to those in need.

Moreover, our findings emphasize the importance of larger centers retrospectively analyzing patient data to enhance patient safety and develop protocols for secure ECMO retrieval. This retrospective analysis enables the identification of potential complications during transport and allows for proactive measures to be implemented, ultimately improving patient outcomes.

## Data availability statement

The raw data supporting the conclusions of this article will be made available by the authors, without undue reservation.

## Ethics statement

The studies involving humans were approved by Integris Baptist Medical Center Institutional Review Board. The studies were conducted in accordance with the local legislation and institutional requirements. The ethics committee/institutional review board waived the requirement of written informed consent for participation from the participants or the participants’ legal guardians/next of kin because The Integris Baptist Medical Center institutional review board approved this study prior to initiation of this project (IRB # 18–005).

## Author contributions

MRM: concept and design, data analysis/interpretation, drafting the article, data collection, and patient care. MOM: concept and design, data interpretation, critical revision of article, and patient care. KC: data collection, critical revision of article, and patient care. LS, RS, JB, AS, CB, and MH: critical revision of article and patient care. AME: data interpretation, critical revision of article, and statistics. AE: concept and design, data interpretation, critical revision of article, approval of article, and patient care. All authors contributed to the article and approved the submitted version.

## Conflict of interest

The authors declare that the research was conducted in the absence of any commercial or financial relationships that could be construed as a potential conflict of interest.

## Publisher’s note

All claims expressed in this article are solely those of the authors and do not necessarily represent those of their affiliated organizations, or those of the publisher, the editors and the reviewers. Any product that may be evaluated in this article, or claim that may be made by its manufacturer, is not guaranteed or endorsed by the publisher.
