# Supplementary material for: Bridging the gap: safety and outcomes of intensivist-led ECMO retrievals
Source: Front Med (Lausanne). 2023 Aug 23;10:1239006. doi: 10.3389/fmed.2023.1239006 (PMC10481738; doi:10.3389/fmed.2023.1239006)
Supplement: Supplementary file 1 [file Data_Sheet_1.docx]

**Supplemental table: Referral criteria**

| **V-V ECMO**   - Severe hypercapnic respiratory failure (Ph < 7.2) - Severe reversible ARDS (PaO2:FiO2 < 100) despite optimal ventilator management, secondary to but not limited to:   - Pneumonia   - Pneumothorax   - Severe asthma   - Inhalation injury - Exclusion criteria:   - Active do not resuscitate   - Chronic pulmonary disease except asthma   - Multi-system organ failure   - Active cardio-pulmonary resuscitation   - Lack of exit strategies | **V-A ECMO**   - SBP less than 90 mmHg/MAP less than 65 mmHg despite two or more vasopressors/inotropes or while on intra-aortic balloon pump or Impella support - Lactic acid > 3 mmol/L - Elevated filling pressures (central venous pressure and/or pulmonary wedge pressure) - Etiology secondary to but not limited to:   - Acute myocardial infarction   - Post cardiotomy cardiogenic shock or inability to wean for cardio-pulmonary bypass   - Acute on chronic heart failure   - Myocarditis   - Postpartum cardiomyopathy   - Post drug intoxication   - Hypothermia   - Malignant arrythmias   - Pulmonary embolism   - High risk catheterization lab procedures - Exclusion criteria:   - Active do not resuscitate   - Irreversible multi-system organ failure   - Lactic acid > 18 mmol/L   - Lack of exit strategies |
| --- | --- |

*V-V ECMO – veno-venous extracorporeal membrane oxygenation, V-A ECMO – veno-arterial extracorporeal membrane oxygenation, ARDS – acute respiratory distress syndrome
